# Hospital Indoor Air as a Reservoir of Opportunistic Filamentous Fungi: Species Diversity and Antifungal Susceptibility

**DOI:** 10.1007/s00284-026-04818-0

**Published:** 2026-03-07

**Authors:** Jhully Pimentel, Glaucia Queiroz dos Santos, Thiago Oliveira Condé, Simone Bravim Maifrede, Thaís Oliveira Scardua, Rubia Miossi, Tatiana Alves dos Reis, Kelly Ishida, Creuza Rachel Vicente, Sarah Santos Gonçalves

**Affiliations:** 1 Center for Research in Medical Mycology (CIMM), Health Sciences Centre (CCS), Federal University of Espírito Santo (UFES), 1468, Marechal Campos Avenue, Vitória, Espírito Santo 29.040-090 Brazil; 2 Infectious Diseases Postgraduate Program, Health Sciences Centre (CCS), Federal University of Espírito Santo (UFES), Vitória, Espírito Santo Brazil; 3 Pathology Department, Health Sciences Centre (CCS), Federal University of Espírito Santo (UFES), Vitória, Espírito Santo Brazil; 4 Infectious Diseases Centre of the Cassiano Antonio de Moraes University Hospital (HUCAM), Vitória, Brazil; 5 Department of Microbiology, Institute of Biomedical Sciences, University of São Paulo (USP), São Paulo, Brazil

**Keywords:** Environmental surveillance, Cryptic species, Azole resistance, air-conditioning systems

## Abstract

Filamentous fungi are common airborne contaminants in hospitals, posing significant risks to immunocompromised patients. This study assessed the diversity, seasonal distribution, and antifungal susceptibility of airborne filamentous fungi, with a focus on *Aspergillus* spp., in two inpatient wards of a tertiary hospital in southeastern Brazil. Air samples were collected seasonally from November 2022 to September 2023 using a six-stage Andersen sampler. Fungal isolates were identified based on macro- and micromorphological characteristics, and *Aspergillus* spp. were further characterised by β-tubulin gene sequencing. In vitro susceptibility to amphotericin B (AMB), itraconazole (ITR), voriconazole (VOR), posaconazole (POS), and anidulafungin (AFG) was evaluated following CLSI guidelines. A total of 583 filamentous fungi were isolated, with *Penicillium*, *Cladosporium* and *Aspergillus* as the most prevalent genera. Fungal concentrations were higher in humidified air and decreased after installation and maintenance of the air-conditioning system. Fourteen *Aspergillus* species were identified across eight sections, including emerging and cryptic taxa such as *A*. *subramanianii*, *A*. *sclerotiorum* and *A*. *alboviridis*. Resistance to ITR was detected in one isolate of *A*. *fumigatus* and one of *A*. *flavus*, while isolates from emerging species of the section *Circumdati* exhibited elevated MICs for AMB and triazoles. All isolates demonstrated low AFG MECs. The study highlights the importance of routine environmental surveillance and antifungal susceptibility testing in hospital settings. The detection of potentially resistant *Aspergillus* species in hospital air emphasises the need to enhance ventilation, monitoring, and diagnostic capabilities to mitigate the risks of healthcare-associated fungal infections.

## Introduction

Filamentous fungi are ubiquitous microorganisms that occur in both outdoor and indoor environments, including residential areas and healthcare facilities. They can colonise abiotic surfaces and are frequently detected in air and water systems [1]. Although many species are saprophytic, certain filamentous fungi display opportunistic behaviour and can cause invasive fungal infections (IFIs), particularly in immunocompromised individuals [2, 3]. Once inhaled, these fungi can colonise the respiratory tract and invade deeper tissues. In more severe cases, they may disseminate through the bloodstream to multiple organs, leading to systemic infections with high mortality rates —a hallmark of many opportunistic fungal pathogens [4, 5].

IFIs are a growing concern in nosocomial settings, particularly in tertiary care hospitals, where inadequate air-conditioning systems and poor hygiene practices can facilitate the spread of fungal conidia. Such conditions heighten the risk of infection in vulnerable patient populations, including those with neutropenia, recipients of hematopoietic stem cell transplants (HSCT), individuals undergoing chemotherapy, and patients receiving prolonged corticosteroid therapy [6]. Patients with chronic conditions, such as chronic obstructive pulmonary disease (COPD) or cystic fibrosis (CF), as well as those with a history of recent invasive procedures or trauma, are also at increased risk [7]. The most common filamentous fungi implicated in these infections include species of the genera *Aspergillus* and *Fusarium*, as well as members of the order Mucorales, with *Aspergillus* spp. being the most frequently reported [8].

In recent years, the prevalence of cryptic and emerging *Aspergillus* species has increased. These organisms frequently exhibit reduced in vitro susceptibility to available antifungal agents, posing significant challenges for treatment. This trend has raised concerns among public health authorities, given the therapeutic challenges and the potential severity of the associated clinical manifestations [9–12].

The microbiological quality of air in hospital environments is a critical determinant in preventing IFIs. This quality can be influenced by several factors, including the presence and maintenance of air-conditioning systems, the type and efficiency of air filters, and the stringency of cleaning and disinfection protocols [13]. Accurate identification of airborne fungal species is essential because different taxa display distinct patterns of virulence and antifungal resistance [14, 15].

In this context, the present study aimed to investigate the diversity and distribution of filamentous fungi in the indoor air of two hospital wards in a university hospital located in southeastern Brazil. The results are expected to yield relevant data on the microbiological quality of hospital air, supporting the implementation of more effective prevention strategies and strengthening epidemiological surveillance, particularly by identifying clinically relevant *Aspergillus* species and evaluating their antifungal susceptibility profiles.

## Materials and methods

### Study site

This study was conducted between November 2022 and September 2023 in two inpatient wards of a tertiary care hospital located in Vitória, Espírito Santo, Brazil. The hospital has a capacity of 277 beds, including a 16-bed Intensive Care Unit (ICU), and provides healthcare services to patients from across the state and neighbouring regions. The selected wards provide care for critically ill patients from multiple medical specialities, including haematology, infectious diseases, cardiovascular surgery, internal medicine, neurology, pulmonology, rheumatology, and cardiology. Both wards follow standard precautions and implement transmission-based precautions—contact, droplet, and airborne—when indicated.

The two wards are located on different floors, each comprising 13 shared rooms. On the 2nd floor, each room measures 26.25 m² (bathroom: 3.32 m²), whereas on the 4th floor, rooms measure 26.73 m² (bathroom: 3.25 m²). All rooms accommodated three beds and were climate-controlled as follows:

- 2nd floor: from November 2022 to January 2023, natural ventilation (windows); from March to September 2023, Split-type air conditioning system.
- 4th floor: climate-controlled with a Split-type air conditioning system throughout the study period.

### Air Sampling in the Wards

At the start of the study, one room was randomly selected on each floor for air sampling. Collections were made in both the inpatient room and the corresponding bathroom. In bathrooms, two conditions were evaluated: dry air and humid air, the latter achieved by running the shower for 5 min before sampling. For clarity in the data analysis, the sampling sites were coded as follows:

- 2nd floor ward: room 2 (Q2), bathroom with dry air (Q2BS), and bathroom with humid air (Q2BU).
- 4th floor ward: room 4 (Q4), bathroom with dry air (Q4BS), and bathroom with humid air (Q4BU).

Air samples were consistently carried out in the morning, between 8 am and 12 pm, twice in different months per season, resulting in a total of eight collections per sampling point (November and December/Spring: C1-C2; January and March/Summer: C3-C4; May and June/Autumn: C5-C6; August and September/Winter: C7-C8). At each sampling event, the ambient temperature and relative humidity were recorded using a digital thermo-hygrometer (INS-1350, Rohs). Airborne fungal particles were collected using a six-stage Andersen-type air sampler (Energética, Brazil) operating at a flow rate of 28.3 L/min. The device was positioned at a height of 1.50 m from the floor, centred in each room and bathroom. For each sampling event, six glass Petri dishes (90 × 15 mm) containing Sabouraud Dextrose Agar (SDA; Oxoid, England) supplemented with 0.5% chloramphenicol were exposed for 30 min, following the manufacturer’s instructions.

After collection, SDA plates were incubated at 25 °C and examined daily for up to 10 days. Plates exhibiting fungal growth were quantitatively analysed by counting colony-forming units (CFUs) and subcultured onto Potato Dextrose Agar (PDA) for identification. Fungal colonies identified as *Aspergillus* were preserved in the microorganism collection of the Centre for Medical Mycology Investigations (CIMM) at the Federal University of Espírito Santo (UFES), Brazil.

### Phenotypic identification of filamentous fungi

Phenotypic identification was based on the assessment of both macro- and micromorphological characteristics. Reproductive fungal structures were examined using a light microscope equipped with a 40x objective (Leica^®^ DM500, Brazil), and identification was performed according to the taxonomic keys described [16].

### Molecular identification and phylogenetic analysis of *Aspergillus* isolates

All *Aspergillus* isolates initially identified phenotypically were subsequently analysed by molecular methods, including Sanger sequencing, for species discrimination. Genomic DNA was extracted directly from the fungal colonies using the PrepMan reagent protocol (Applied Biosystems, USA). DNA concentration and purity were determined using a NanoVue Plus spectrophotometer (GE Healthcare, USA). The partial β-tubulin (*BenA*) gene region was amplified using the forward primer BT2a (5′-GGT AAC CAA ATC GGT GCT GCT TTC-3′) and reverse primer BT2b (5′-ACC CTC AGT GTA GTG ACC CTT GGC-3′) [17]. Each PCR reaction was carried out in a final volume of 25 µL, containing 12.5 µL of PCR Master Mix^®^ (Promega, USA), 2 µL of each primer at 20 pmol/µL (Invitrogen, USA), 2 µL of DNA (40 ng/µL), and 6.5 µL of Milli-Q water.

The PCR amplification program consisted of an initial denaturation at 94 °C for 3 min, followed by 40 cycles of denaturation at 94 °C for 1 min, annealing at 55 °C for 1 min, and extension at 72 °C for 1 min. The final extension step was performed at 72 °C for 5 min. PCR products were purified using the QIAquick PCR Purification Kit (Qiagen, Germany) according to the manufacturer’s instructions and stored at − 20 °C until sequencing. Sequencing was performed in both directions using the BigDye™ Terminator v3.1 Cycle Sequencing Kit (Applied Biosystems, USA). Capillary electrophoresis sequencing was carried out on an ABI 3730 DNA Analyzer (Thermofisher/Applied Biosystems, USA). Sequence data were obtained using AutoAssembler and Sequencing Analysis Software (Thermofisher/Applied Biosystems, USA), and subsequent editing and alignment were performed using Sequencher DNA Sequence Assembly Software v4.1.4 (Gene Codes Corporation, USA).

Initially, BLASTn (https://blast.ncbi.nlm.nih.gov/Blast.cgi*)* was used to identify *Aspergillus* isolates at the section rank using the *BenA* sequences generated in this study. Subsequently, a dataset containing *BenA* sequences was constructed using the newly generated sequences, ex-type sequences, and reference sequences of *Aspergillus* available in GenBank. DNA alignment was performed using MAFFT v. 7 [18] with the L-INS-I option, and manual adjustments were performed in MEGA v. 7 [19]. A maximum-likelihood (ML) phylogenetic tree was constructed using IQTREE v. 2.4.0 [20], with 10,000 ultrafast bootstrapping (UFBoot) replicates. The best-fit nucleotide substitution model was determined using ModelFinder based on the corrected Akaike Information Criterion (AICc). The phylogenetic tree was rooted with *Penicillium digitatum* CBS 112,082 and *Penicillium expansum* CBS 325.48. Only UFBoot support values equal to or higher than 70 were plotted at the nodes. Phylogenetic trees were visualised using FigTree v. 1.4.3 [21] and then exported to Microsoft PowerPoint v. 2507 for further editing.

The DNA sequences generated in this study were deposited in GenBank (accession numbers: PX148699–PX148725) (https://www.ncbi.nlm.nih.gov/genbank/*).*

### In vitro antifungal susceptibility testing of *Aspergillus* isolates

The in vitro antifungal susceptibility of *Aspergillus* spp. isolates was determined using the broth microdilution method, following the Clinical and Laboratory Standards Institute (CLSI) M38-A2 guidelines. Antifungal agents were obtained from Sigma Chemical (USA), except for itraconazole, which was supplied by Janssen Pharmaceutica (Belgium). The drug concentrations ranged from 16 to 0.03 µg/mL. The reference strain *Aspergillus flavus* ATCC™ 204,304™ was included as a quality control in all assays [22, 23].

Susceptibility results were interpreted according to the epidemiological cutoff values (ECVs) established in the CLSI M59 document [24] for itraconazole (ITR), voriconazole (VOR), posaconazole (POS), and amphotericin B (AMB), except for anidulafungin (AFG). The minimum inhibitory concentration (MIC) was defined as the lowest antifungal concentration that completely inhibited visible fungal growth. In contrast, the minimum effective concentration (MEC) of AFG was defined as the lowest concentration that induced morphological alterations in fungal hyphae.

### Data Analysis

Quantification of airborne fungi and physical parameters was performed using descriptive statistics, including minimum, maximum, median, and geometric mean values, in Microsoft Excel 2016. The data were subsequently exported to SPSS^®^ version 20 (IBM^®^, USA) for statistical analysis and GraphPad Prism (GraphPad Software, USA) for graphical representation.

## Results

### Airborne fungal quantification on the second and fourth floors

**Fig. 1:**
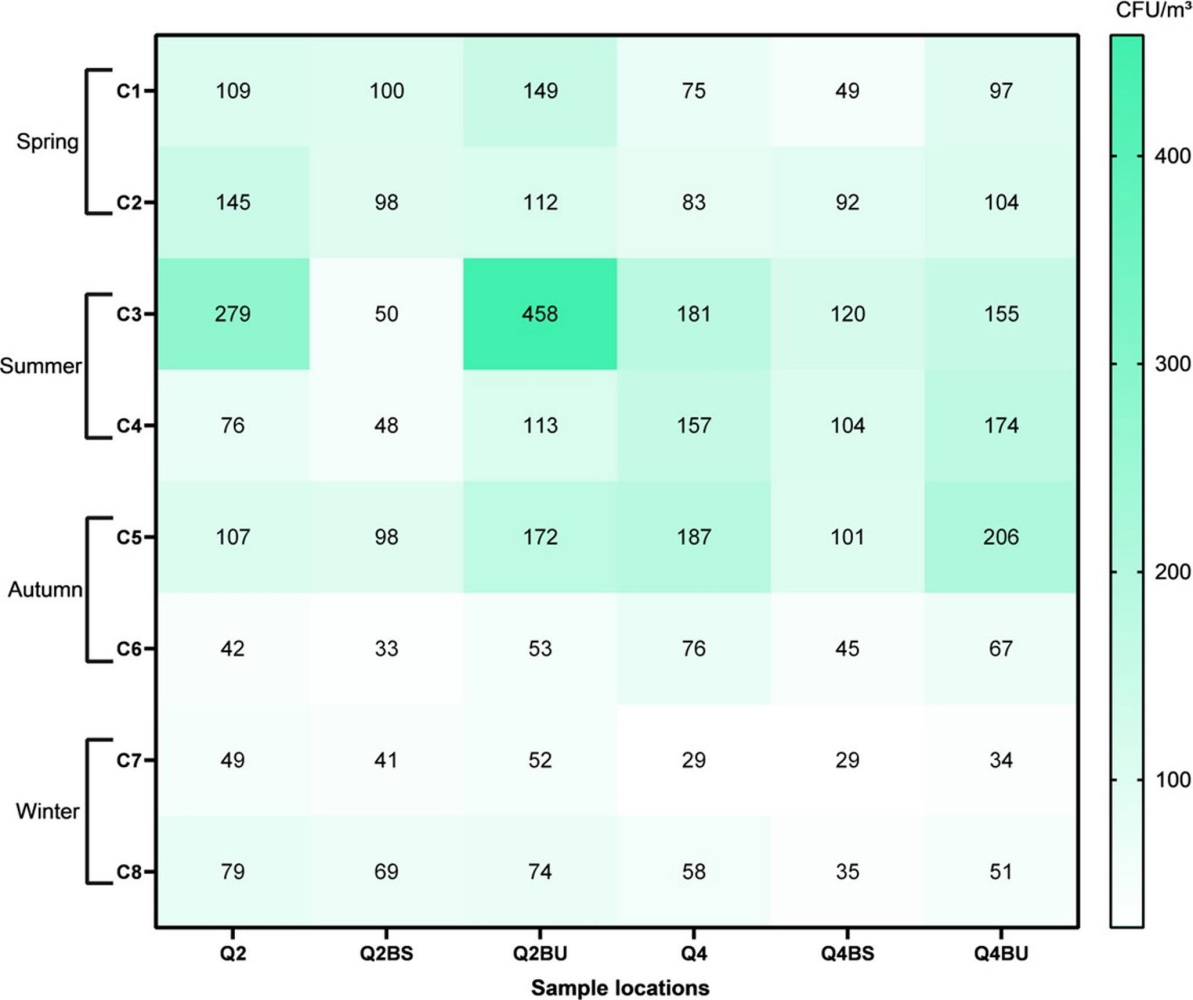
Absolute concentrations of airborne fungi (CFU/m³) in the wards and their respective bathrooms (dry and humid air) during sampling on **(A)** the 2nd floor and **(B)** the 4th floor. 1st sampling (C1). 2nd sampling (C2). 3rd sampling (C3). 4th sampling (C4). 5th sampling (C5). 6th sampling (C6). 7th sampling (C7). 8th sampling (C8); 2nd floor – room (Q2). dry bathroom (Q2BS). humid bathroom (Q2BU)

On the second floor, total airborne fungal concentrations ranged from 33 CFU/m³ to 458 CFU/m³ across all sampling events and sites (Fig. 1). The highest counts at Q2 and Q2BU were observed during C3 (279 and 458 CFU/m³, respectively), whereas Q2BS was in C1 (100 CFU/m³). This floor changed ventilation, transitioning from natural ventilation (C1–C3) to split-type air conditioning (C4–C8).

After air conditioning installation (C4–C8), concentrations decreased to 33–172 CFU/m³, with the highest total count in C5 (377 CFU/m³) and the most significant difference between Q2BS and Q2BU (74 CFU/m³). The lowest values were observed in C6 and C7, with C6 exhibiting the lowest total value (128 CFU/m³). The median values dropped to 76, 48, and 74 CFU/m³ at Q2, Q2BS, and Q2BU, respectively. Increases in CFU/m³ in humid-air bathrooms compared to dry-air ranged from 7.2% to 75.5%, depending on the sampling event.

On the fourth floor, where air conditioning was in use throughout the study, airborne fungal concentrations ranged from 29 to 206 CFU/m³ (Fig. 1). The highest values were recorded in C5 at Q4 (187 CFU/m³), Q4BS (101 CFU/m³), and Q4BU (206 CFU/m³). The most significant variation occurred in C5 (101–206 CFU/m³), which also showed the highest total count (494 CFU/m³). The lowest total and point values were observed in C7 (92 CFU/m³ total; 29–34 CFU/m³ per site).

Fungal concentrations were consistently higher in humid-air bathrooms compared to dry-air conditions, particularly in C1 (98%) and C5 (104%). From C6 onward, a general decline was observed. Although the ventilation type remained unchanged, the air-conditioning unit was cleaned after C5, leading to a notable reduction in the median CFU/m³: from 157, 101, and 155 (C1–C5) to 58, 35, and 51 (C6–C8) at Q4, Q4BS, and Q4BU, respectively.

### Indoor atmospheric parameters: Temperature and relative humidity

The atmospheric conditions varied across the sampling events (Table 1). The relative humidity (RH) ranged from 43% to 96%, with C1 showing the highest (72–96%) and C4 the lowest values (43–80%). Greater RH variability was observed on the second floor (Q2 and Q2BS) compared to the fourth floor (Q4 and Q4BS), reflecting the impact of changing ventilation conditions. The RH values were generally higher in humidified bathrooms (Q2BU and Q4BU), with C1 reaching the highest RH (92% at Q2BU and 96% at Q4BU).

**Table 1:** Atmospheric temperature (°C) and air relative humidity (%) during air sampling at the collection points included in the study

Sampling	Sampling Points												Min./Max.	
	Q2		Q2BS		Q2BU		Q4		Q4BS		Q4BU			
	°C	%	°C	%	°C	%	°C	%	°C	%	°C	%	°C	%
C1	26.2	79	26.8	87	25.1	92	26.1	76	24.3	72	24.3	96	24.3–26.2	72–96
C2	27.7	71	29.9	69	29.9	79	24.3	64	29.2	67	24.1	86	24.1–29.9	64–86
C3	28.4	69	28.7	87	27.7	89	28.2	76	30.5	76	28	90	27.7–30.5	69–90
C4	23.3	43	23.9	59	24.6	78	26.7	72	27.5	80	26.5	85	23.3–27.5	43– 80
C5	24.4	50	25.7	67	24.5	91	25.5	73	26	72	25.2	89	24.4–25.7	50– 91
C6	26.2	65	25	58	24.8	76	25	68	25.6	66	25	90	24.8–26.2	58– 90
C7	27.5	63	28.1	64	27.4	80	27.3	73	27.5	78	26.6	79	26.6–28.1	63–80
C8	29.4	59	29	62	29	70	28.4	62	28.6	72	27.2	81	27.2–29.4	59–81
Min./Max.	23.3–29.4	43–79	23.9–29.9	58–87	24.5–29.9	70–92	24.3–28.4	62–76	24.3–30.5	66–80	24.3–28	79–96	-	-

‘-‘: not observation/not measured; (C): Sampling; 1st sampling (C1). 2nd sampling (C2). 3rd sampling (C3). 4th sampling (C4). 5th sampling (C5). 6th sampling (C6). 7th sampling (C7). 8th sampling (C8); 2nd floor - room (Q2). Dry bathroom (Q2BS). humid bathroom (Q2BU); 4th floor - room (Q4). dry bathroom (Q4BS). Humid bathroom (Q4BU)

In the bathrooms, RH variation was more pronounced on the second floor (Q2BS: 29%; Q2BU: 22%) than on the fourth floor (Q4BS: 14%; Q4BU: 17%), indicating a greater influence of humidification. The temperature ranged from 23.3 °C to 30.5 °C, with the widest variation in C2 (24.1–29.9 °C) and the narrowest in C5 (24.4–25.7 °C). The second-floor room (Q2) tended to present slightly higher temperatures than Q4. The differences between dry- and humid-air bathrooms were minor, with somewhat greater temperature variation in dry-air conditions. Overall, the temperature remained relatively stable throughout the study.

### Identification of filamentous fungi

**Fig. 2:**
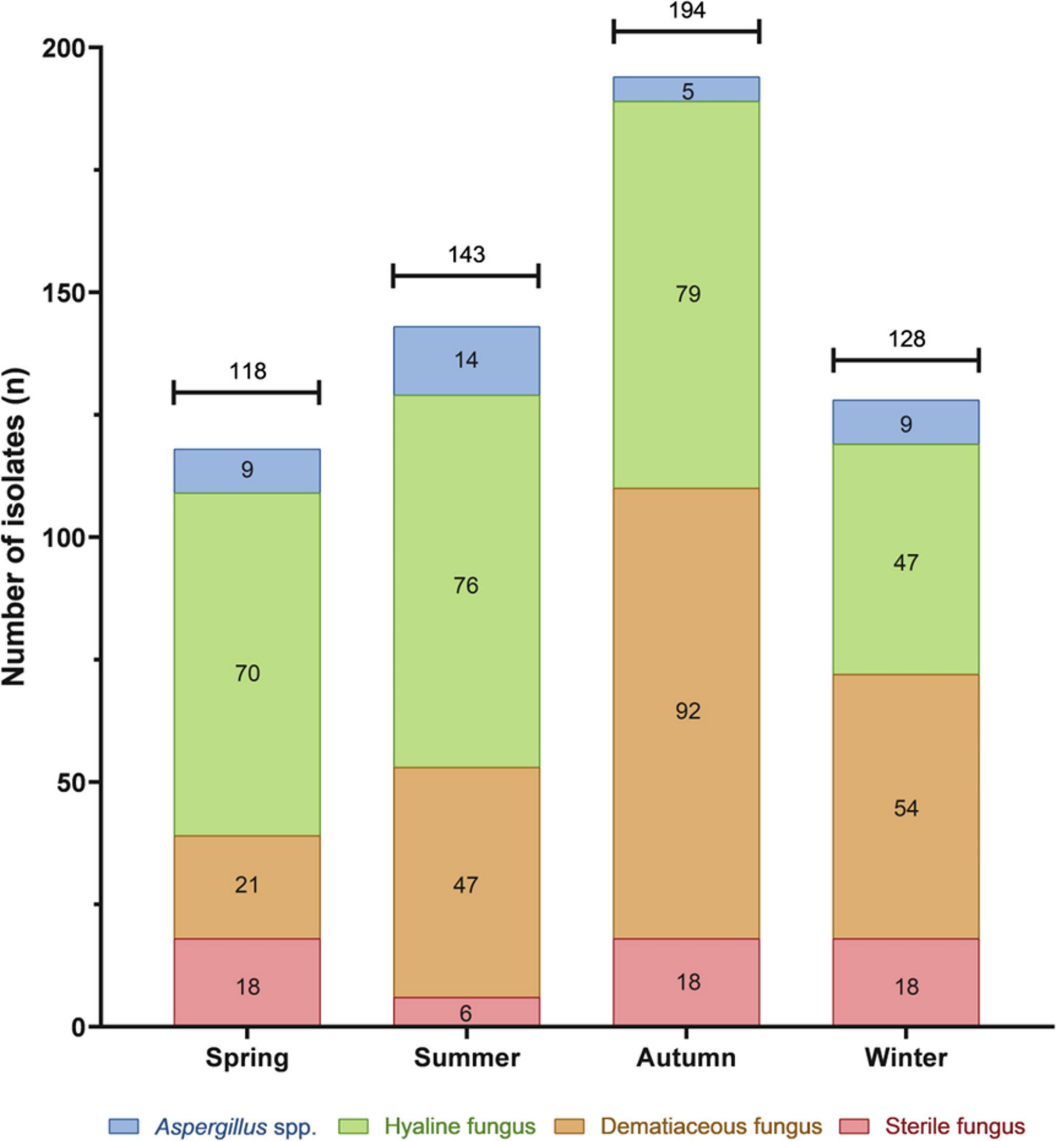
Occurrence of filamentous fungi in the indoor air of the wards (including rooms and bathrooms) from November 2022 to September 2023

A total of 583 filamentous fungal isolates were recovered from indoor air samples collected throughout the seasons (Fig. 2) and grouped into four categories: *Aspergillus* spp., hyaline fungi, dematiaceous fungi, and sterile fungi. Hyaline fungi were the most frequently recovered group (272 isolates; 46.7%), with higher occurrences in autumn (79; 13.5%) and summer (76; 13%), respectively. Dematiaceous fungi were the second most prevalent (214 isolates; 36.7%), with a peak in autumn (92; 15.8%). A total of 37 isolates of *Aspergillus* spp. (6.3%) were obtained, with the highest number recorded in summer (14; 2.4%). Sterile fungi accounted for 60 isolates (10.3%), with relatively stable distribution across seasons.

Seventeen fungal genera were identified during the sampling period. Autumn showed the highest diversity, with 13 genera (76.5%), while winter had the lowest, with eight genera (47.1%). Among the sampling sites, Q2 (second floor) exhibited the most incredible diversity across seasons (6–7 genera per season), whereas Q4 and Q4BS (fourth floor) showed peak diversity in autumn (8 genera each).

Regarding the isolate counts, the humidified bathroom Q2BU recorded the highest number of fungal isolates (111/583; 19.0%), while the dry bathroom Q2BS had the lowest (104/583; 17.8%). On the fourth floor, the Q4 room yielded 93 isolates (15.9%), followed by Q4BU with 77 isolates (13.2%).

All the recovered genera are considered opportunistic pathogens (Table 2). The most frequently isolated genus was *Penicillium* (225/583; 38.6%), which was consistently present in all seasons (40–63 isolates), followed by *Cladosporium* (169/583; 30.0%) and *Aspergillus* (37/583; 6.4%). *Aspergillus* was most frequently isolated during summer (14 isolates) and least in autumn (5 isolates). In spring, Q2 yielded the highest number of *Aspergillus* isolates (*n* = 4). The less prevalent genera included *Curvularia* (0.7%), *Lomentospora* (0.3%), *Alternaria* (0.2%), and *Fusarium* (0.2%).

**Table 2:** Occurrence of fungal genera found in air samples collected at different points in the wards during the seasons

Season	Identification	Sampling Points (*n*/%)						
		Q2	Q2BS	Q2BU	Q4	Q4BS	Q4BU	Total
Spring	Genus (*n* = 10)	6	6	7	5	3	4	31
	*Penicillium*	15/55.6	15/65.2	9/40.9	11/52.4	7/46.7	5/50.0	62/52.5
	*Aspergillus*	4/14.8	1/4.3	2/9.1	1/4.8	-	1/10.0	9/7.6
	*Cladosporium*	3/11.1	4/17.4	6/27.3	2/9.5	3/20.0	-	18/15.3
	*Colletotrichum*	1/3.7	-	-	-	-	-	1/0.8
	*Paecilomyces*	-	1/4.3	1/4.5	-	-	-	2/1.7
	*Acremonium*	-	1/4.3	-	1/4.8	-	-	2/1.7
	*Exophiala*	-	-	1/4.5	-	-	1/10.0	2/1.7
	*Purpureocillium*	1/3.7	-	-	-	-	-	1/0.8
	*Curvularia*	-	-	1/4.5	-	-	-	1/0.8
	Sterile Fungus	2/7.4	1/4.3	1/4.5	6/28.6	5/33.3	3/30.0	18/15.3
	Hyaline no-identified	1/3.7	-	1/4.5	-	-	-	2/1.7
	Total	27/100	23/100	22/100	21/100	15/100	10/100	118/100
Summer	Genus (*n* = 10)	7	5	5	5	6	7	35
	*Penicillium*	5/26.3	9/40.9	13/41.9	11/45.8	16/61.5	9/42.9	63/44.1
	*Aspergillus*	2/10.5	1/4.5	3/9.7	2/8.4	3/11.5	3/14.3	14/9.8
	*Cladosporium*	2/10.5	7/31.8	9/29.0	4/16.7	4/15.4	5/23.8	31/21.7
	*Fonsecaea*-like	4/21.1	-	2/6.5	2/8.3	1/3.8	1/4.8	10/7.0
	*Paecilomyces*	3/15.8	3/13.6	3/9.7	-	1/3.8	-	10/7.0
	*Trichoderma*	-	-	-	-	-	1/4.8	1/0.7
	*Phoma*	-	-	-	-	-	1/4.8	1/0.7
	*Torula*	1/5.3	-	-	-	-	-	1/0.7
	*Curvularia*	1/5.3	-	-	-	-	-	1/0.7
	Sterile Fungus	-	2/9.1	-	2/8.3	1/3.8	1/4.8	6/4.2
	Hyaline no-identified	1/5.3	-	-	1/4.2	-	-	2/1.4
	Dematiaceous no-identified	-	-	1/3.2	2/8.3	-	-	3/2.1
	Total	19/100	22/100	31/100	24/100	26/100	21/100	143/100
Autumn	Genus (*n* = 13)	7	6	4	8	8	7	40
	*Penicillium*	8/27.6	11/33.3	14/38.9	11/32.4	9/25.7	7/25.9	60/31.0
	*Aspergillus*	1/3.4	-	-	3/8.8	-	1/3.7	5/2.6
	*Cladosporium*	15/51.7	15/45.5	12/33.3	9/26.5	10/28.6	8/29.6	69/35.6
	*Fonsecaea-*like	1/3.4	1/3.0	6/16.7	2/5.9	3/8.6	3/11.1	16/8.2
	*Paecilomyces*	-	1/3.0	1/2.8	1/2.9	-	-	3/1.5
	*Trichoderma*	-	-	-	-	-	1/3.7	1/0.5
	*Torula*	-	1/3.0	-	-	-	-	1/0.5
	*Acremonium*	1/3.4	-	-	-	2/5.7	2/7.4	5/2.6
	*Lomentospora*	1/3.4	-	-	-	1/2.9	-	2/1.0
	*Purpureocillium*	-	-	-	1/2.9	-	-	1/0.5
	*Fusarium*	-	-	-	-	1/2.9	-	1/0.5
	*Curvularia*	-	-	-	1/2.9	1/2.9	-	2/1.0
	Sterile Fungus	2/6.9	1/3.0	-	3/8.8	7/20.0	5/18.5	18/9.3
	Hyaline no-identified	-	2/6.1	2/5.6	2/5.9	-	-	6/3.1
	Dematiaceous no-identified	-	1/3.0	1/2.8	1/2.9	1/2.9	-	4/2.1
	Total	29/100	33/100	36/100	34/100	35/100	27/100	194/100
Winter	Genus (*n* = 8)	7	4	6	5	4	5	31
	*Penicillium*	6/18.2	8/30.8	6/27.3	7/50.0	6/42.9	7/36.8	40/31.3
	*Aspergillus*	3/9.1	2/7.7	-	1/7.1	-	3/15.8	9/7.0
	*Cladosporium*	17/51.5	12/46.2	9/40.9	4/28.6	3/21.4	6/31.6	51/39.8
	*Fonsecaea*-like	1/3.0	-	-	1/7.1	-	-	2/1.6
	*Paecilomyces*	1/3.0	-	1/4.5	-	-	-	2/1.6
	*Alternaria*	-	-	1/4.5	-	-	-	1/0.8
	*Purpureocillium*	1/3.0	-	1/4.5	-	1/7.1	1/5.3	4/3.1
	Sterile Fungus	4/12.1	4/15.4	3/13.6	1/7.1	4/28.6	2/10.5	18/14.1
	Hyaline no-identified	-	-	1/4.5	-	-	-	1/0.8
	Total	33/100	26/100	22/100	14/100	14/100	19/100	128/100
The total number in a year		108	104	111	93	90	77	583

n − number of isolates. 2nd floor − room (Q2). dry bathroom (Q2BS). humid bathroom (Q2BU); 4th floor − room (Q4). dry bathroom (Q4BS). humid bathroom (Q4BU); ‘−‘: not observation/not measured

### Polyphasic characterisation of *Aspergillus* isolates

Of the 37 *Aspergillus* isolates recovered during the study, 27 were viable for macro- and micromorphological identification and phylogenetic analysis of the β-tubulin (*BenA*) gene region. The phylogenetic analysis of the *BenA* alignment contained 62 sequences, and the length comprised 531 characters (including gaps) of which 205 were constant sites, 309 were parsimony-informative sites, and 17 were singletons. The best-fit nucleotide substitution model chosen was TPM3 + I+G4.

**Fig. 3:**
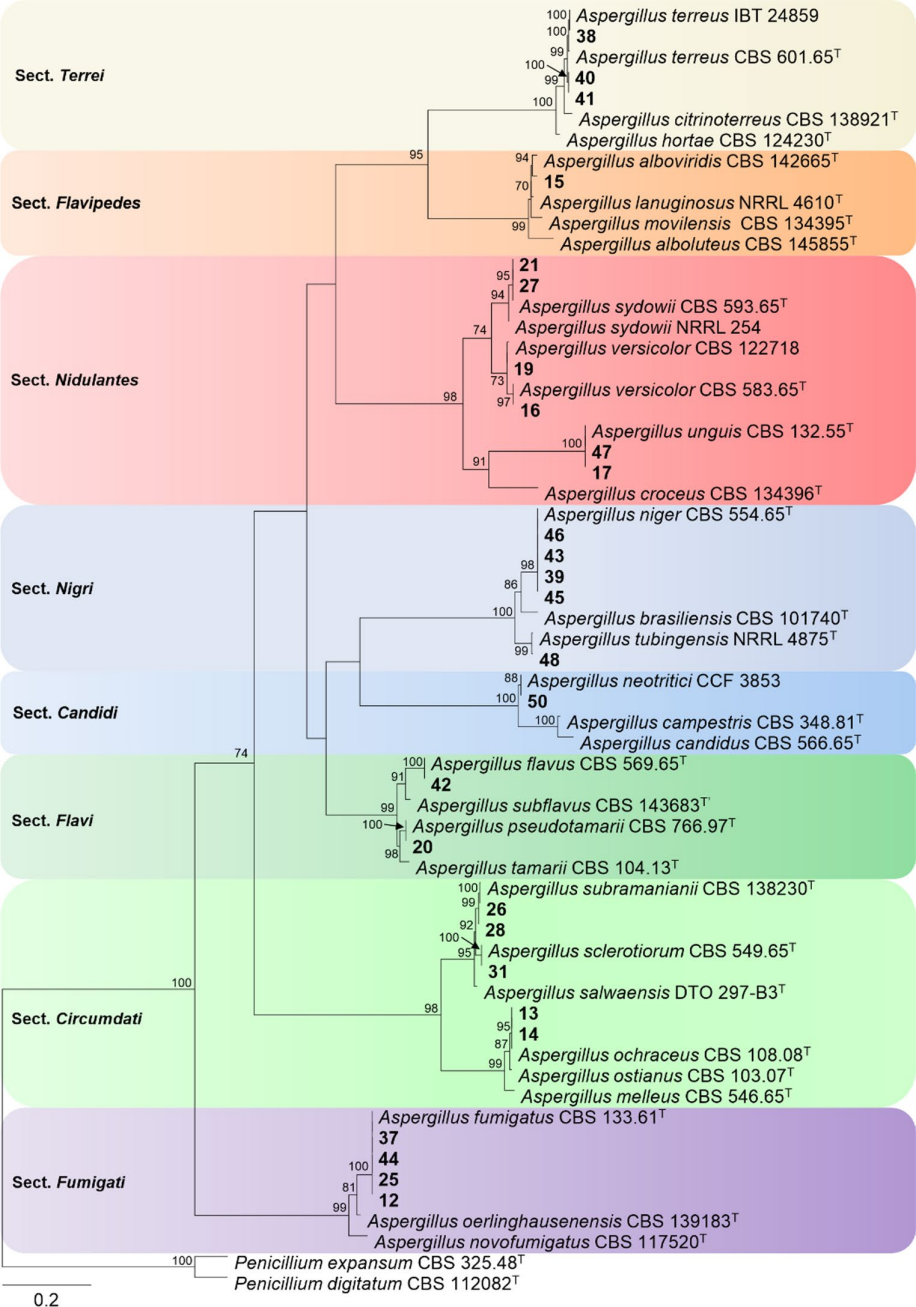
Maximum-likelihood phylogenetic tree of *Aspergillus* species based on the partial *BenA* gene region. Isolates recovered in this study are shown in bold. Ex-type isolates are marked with ‘‘T.’’ Only bootstrap (bs) values ≥ 70% are shown at branches. The tree was rooted with *Penicillium digitatum* CBS 112,082 and *Penicillium expansum* CBS 325.48

Based on these results, 14 *Aspergillus* species distributed across eight sections were identified (Fig. 3). In the *Nidulantes* section, six isolates were clustered into three species: *A*. *sydowii* (*n* = 2), *A. unguis* (*n* = 2), and *A*. *versicolor* (*n* = 2) (Fig. 3). The *Circumdati* section comprised five isolates distributed in *A. ochraceus* (*n* = 2), *A. subramanianii* (*n* = 2), and *A. sclerotiorum* (*n* = 1). In the *Nigri* section, five isolates were clustered into two species, *A. niger* (*n* = 4) and *A. tubingensis* (*n* = 1). Other sections identified included *Terrei* with *A. terreus* (*n* = 3), *Flavi* with *A. flavus* (*n* = 1) and *A. pseudotamarii* (*n* = 1), *Fumigati* with *A. fumigatus* (*n* = 4), *Flavipedes* with *A. alboviridis* (*n* = 1), and *Candidi* with *A. neotritici* (*n* = 1).

### In vitro susceptibility profile of *Aspergillus* isolates to azoles, polyene, and echinocandin antifungals

Twenty-seven *Aspergillus* isolates were tested for in vitro susceptibility to AMB, ITR, POS, VOR, and AFG according to CLSI guidelines (Table 3).

**Table 3:** In vitro susceptibility profile of 27 isolates of *Aspergillus* spp. to amphotericin B, itraconazole, voriconazole, posaconazole, and anidulafungin by the broth microdilution method according to the CLSI protocol

Species (n)	Antifungal	ECV (µg/mL)	MIC/MEC (µg/mL)			Number of isolates with MIC/MEC value (µg/mL):										
			Variation of MIC	MG	NW (n)	0.03	0.06	0.125	0.25	0.5	1	2	4	8	16	16>
*Aspergillus niger* (4)	AMB	2	0.25–0.5	0.42	0	-			1	3						
	ITRA	4	2	2	0							4				
	VOR	2	0.5	0.5	0					4						
	POS	2	0.5	0.5	0					4						
	AFG	-	0.03–0.5	0.07	NE	2	1			1						
*Aspergillus fumigatus* (4)	AMB	2	1	1.00	0						4					
	ITRA	1	0.5– 2	1.00	1					1	2	1				
	VOR	1	0.25–1	0.35	0				3		1					
	POS	-	0.25–1	0.31	NE				3		1					
	AFG	-	0.03	0.03	NE	4										
*Aspergillus terreus* (3)	AMB	4	0.5–2	1.26	0					1		2				
	ITRA	2	0.5–1	0.79	0					1	2					
	VOR	2	0.5–1	0.63	0					2	1					
	POS	1	0.25–0.5	0.40	0				1	2						
	AFG	-	0.03	0.03	NE	3										
*Aspergillus ochraceus* (2)	AMB	-	4	4	NE								2			
	ITRA	-	1	1	NE						2					
	VOR	-	0.5	0.5	NE					2						
	POS	-	1	1	NE						2					
	AFG	-	0.03	0.03	NE	2										
*Aspergillus subramanianii* (2)	AMB	-	4	4	NE								2			
	ITRA	-	2–4	2.83	NE							1	1			
	VOR	-	2	2	NE							2				
	POS	-	2	2	NE							2				
	AFG	-	0.03	0.03	NE	2										
*Aspergillus unguis* (2)	AMB	-	1–2	1.41	NE						1	1				
	ITRA	-	2–4	2.83	NE							1	1			
	VOR	-	0.25–0.5	0.35	NE				1	1						
	POS	-	0.5–2	1	NE					1		1				
	AFG	-	0.03	0.03	NE	2										
*Aspergillus sydowii* (2)	AMB	-	1–2	1.41	NE						1	1				
	ITRA	-	1	1	NE						2					
	VOR	-	0.5–1	0.71	NE					1	1					
	POS	-	0.5	0.5	NE					2						
	AFG	-	0.03	0.03	NE	2										
*Aspergillus versicolor* (2)	AMB	2	1	1	0						2					
	ITRA	-	1	1	NE						2					
	VOR	-	0.25	0.25	NE				2							
	POS	-	0.25–0.5	0.35	NE				1	1						
	AFG	-	0.03	0.03	NE	2										
*Aspergillus sclerotiorum* (1)	AMB	-	2	-	NE							1				
	ITRA	-	> 16	-	NE											1
	VOR	-	2	-	NE							1				
	POS	-	2	-	NE							1				
	AFG	-	0.03	-	NE	1										
*Aspergillus tubingensis* (1)	AMB	-	0.25	-	NE				1							
	ITRA	-	2	-	NE							1				
	VOR	-	1	-	NE						1					
	POS	-	0.5	-	NE					1						
	AFG	-	0.5	-	NE					1						
*Aspergillus pseudotamarii* (1)	AMB	-	2	-	NE							1				
	ITRA	-	1	-	NE						1					
	VOR	-	0.25	-	NE				1							
	POS	-	0.5	-	NE					1						
	AFG	-	0.03	-	NE	1										
*Aspergillus flavus* (1)	AMB	4	2	-	0							1				
	ITRA	1	2	-	1							1				
	VOR	2	0.25	-	0				1							
	POS	0.5	2	-	1							1				
	AFG	-	0.03	-	NE	1										
*Aspergillus neotritici* (1)	AMB	-	1	-	NE						1					
	ITRA	-	0.5	-	NE					1						
	VOR	-	0.06	-	NE		1									
	POS	-	0.5	-	NE					1						
	AFG	-	0.03	-	NE	1										
*Aspergillus alboviridis* (1)	AMB	-	2	-	NE							1				
	ITRA	-	2	-	NE							1				
	VOR	-	0.5	-	NE					1						
	POS	-	1	-	NE						1					
	AFG	-	0.03	-	NE	1										

‘−‘: not observation/not measured; n: number of isolates; GM: geometric mean; MIC: minimum inhibitory concentration; MEC: minimum effective concentration; NW: non−wild. potentially resistant. NE: no determined eco value; ECV: epidemiological cutoff; AMB: amphotericin b; ITR: itraconazole; VOR: voriconazole; POS: posaconazole; AFG: anidulafungin

All isolates of *A. flavus*, *A. fumigatus*, *A. niger*, *A. terreus*, and *A. versicolor* were classified as wild-type (WT) for AMB (Table 3). High MIC values for AMB were observed among emerging species from the *Circumdati*, *Flavivipedes*, and *Nidulantes* sections, particularly *A. ochraceus* and *A. subramanianii* (MIC = 4 µg/mL for both).

Non-WT phenotypes for ITR were detected in one isolate of *A. fumigatus* and one of *A. flavus* (MIC > 1 µg/mL), whereas all isolates of *A. niger* and *A. terreus* were considered WT. The *A. sclerotiorum* isolate exhibited a high MIC (> 16 µg/mL) for ITR, while those of *A. unguis* and *A. subramanianii* showed moderately high MICs of 4 µg/mL; however, no ECVs were available for these species. For VOR, isolates of *A. flavus*, *A. fumigatus*, *A. niger*, *A. terreus*, and *A. versicolor* were considered WT. Regarding POS, a non-WT phenotype was observed only in the *A. flavus* isolate (MIC > 1 µg/mL). For AFG, all isolates exhibited low MECs values ranging from 0.03 to 0.5 µg/mL.

## Discussion

This study demonstrated that hospital air harbours an underestimated diversity of filamentous fungi, including emerging and cryptic *Aspergillus* species, some of which exhibited elevated MICs to first-line antifungals. Notably, triazole resistance was detected in *A. fumigatus* and *A. flavus*, and emerging species such as *A. subramanianii* and *A. sclerotiorum* also displayed concerning elevated MICs. These findings underscore the potential risk of hospital-associated fungal infections and highlight the importance of continuous environmental surveillance coupled with antifungal susceptibility testing.

Fungal concentrations in this study ranged from 33 to 458 CFU/m³ on the second floor and from 29 to 187 CFU/m³ on the fourth floor, exceeding levels reported in previous studies using single-stage samplers [25, 26]. This discrepancy may be attributed to the six-stage sampler used in this study, which captures a broader range of particle sizes. Nevertheless, all values remained below the 750 CFU/m³ threshold established by the Brazilian Health Regulatory Agency (ANVISA) [27]. Currently, there are no international guidelines that define universal numerical thresholds for permissible or desirable airborne fungal concentrations in healthcare facilities [27, 28]. The WHO and other international bodies primarily emphasise environmental control strategies, such as adequate ventilation, use of high-efficiency particulate air (HEPA) filtration, infrastructure maintenance, and infection prevention measures, rather than absolute quantitative limits for airborne fungi [13, 27, 29]. Accordingly, fungal air counts should be interpreted in the context of the hospital setting, patient population, ventilation systems, and local epidemiological conditions [13, 30].

The shift from natural to artificial ventilation on the second floor and HVAC cleaning on the fourth floor resulted in substantial reductions in airborne fungal load, particularly at sampling points C4 and C6 onward. These findings are consistent with previous reports demonstrating the benefits of air-conditioning systems and proper HVAC maintenance in controlling fungal bioaerosols [13].

Humidified air consistently led to increased fungal concentrations in bathrooms across all collections. The most notable increase occurred during C3 on the second floor, with an 816% increase compared with dry-air conditions. This trend was also observed on the fourth floor, particularly during C1 and C5. These findings underscore the role of water as a vector for fungal bioaerosol dissemination, linked to biofilm formation in water systems [28, 29], and emphasise the influence of ventilation type, humidity, and HVAC maintenance on the concentration of airborne fungal propagules in hospital settings.

Seventeen fungal genera were identified, with *Penicillium*, *Cladosporium*, and *Aspergillus* being the most prevalent, and diversity was highest in autumn. These genera are well-adapted to airborne dissemination owing to their small, hydrophobic conidia and metabolic flexibility [7, 30]. Their persistence in healthcare settings has been widely documented [31, 32]. In contrast, Mucorales were not recovered in this study, and *Fusarium* was identified only once. Interestingly, 28 isolates were identified as *Fonsecaea*-like based on their macro-and micromorphological characteristics. Members of the family *Herpotrichiellaceae* may exhibit similar asexual structures, which can make proper identification challenging [33]. Further studies using molecular data, such as ITS sequencing, are needed to clarify the identity of these isolates, environmental distribution, and potential clinical relevance of airborne *Fonsecaea*-like fungi in hospital settings.

Using a polyphasic approach, 14 *Aspergillus* species were identified across eight sections, reflecting the underreported fungal diversity in hospital air in Brazil [34, 35]. The most frequently recovered species were *A. fumigatus*, *A. niger*, and *A. terreus*, which are well-known agents of invasive aspergillosis [36, 37].

*Aspergillus* section *Fumigati* was detected at a low frequency in this study, consistent with previous reports [25, 38], but contrary to others who reported it as predominant [34, 39]. All isolates in this section were identified as *A. fumigatus*, a key etiological agent of respiratory fungal diseases [7]. Although *A. nidulans* was not detected, six isolates from the *Nidulantes* section were recovered, which also pose clinical risks [40].

Interestingly, *Circumdati* was the second most frequent section in this study, alongside *Nigri*. This contrasts with previous studies, where *Nigri* typically ranked second in hospital air [34, 38]. Species such as *A. ochraceus*, *A. subramanianii*, and *A. sclerotiorum* are known producers of ochratoxin A and have been linked to cutaneous and invasive infections [41, 42].

The observed antifungal susceptibility profiles demonstrate that resistance to triazoles among *Aspergillus* species is heterogeneous and that cross-resistance is not mandatory. Although non-WT phenotypes for ITR were detected in *A. fumigatus* and *A. flavus*, susceptibility to VOR was preserved across all tested species, indicating that reduced activity of one triazole does not necessarily predict reduced susceptibility to others. Similarly, decreased susceptibility to POS was observed in only one *A. flavus* isolate. Such findings are concerning, given the clinical reliance on triazoles and their widespread use in agriculture [43, 44]. Similar resistance trends have been documented across Europe, Asia, and South America [45, 46].

Emerging species from the *Circumdati* section, including *A. ochraceus*, *A. subramanianii*, and *A. sclerotiorum*, exhibited elevated MICs to ITR and AMB. While the absence of established ECVs precludes formal WT classification, the consistently high MIC values observed raise concerns regarding their antifungal susceptibility profiles and pose challenges in therapeutic decision-making.

Overall, these findings highlight the importance of assessing each triazole individually and reinforce the need for expanded susceptibility data, establishment of ECVs, and improved identification and antifungal testing of underrecognised *Aspergillus* species.

Conversely, most isolates exhibited low MICs for AMB, except for *A. flavus* and *A. terreus*, which have intrinsic resistance [47]. Although AMB resistance is rare, it has been reported [48] and may serve as an alternative in azole-resistant infections, except for intrinsically resistant species [47]. Thus, antifungal susceptibility profiling remains essential.

Finally, echinocandins, particularly anidulafungin, demonstrated promising activity against all isolates, suggesting a potential role in managing triazole-resistant infections [49]. Altogether, these findings reinforce the need for robust species-level identification, expanded susceptibility datasets, and improved antifungal testing strategies for underrecognised *Aspergillus* species [50].

Although airborne fungi were highly prevalent, no fungal infections were diagnosed during the study period. However, the absence of advanced diagnostic tools likely contributed to underdetection, especially in immunocompromised patients. This gap highlights the need to strengthen diagnostic infrastructure and integrate environmental surveillance with clinical data in resource-limited settings.

## Conclusion

Despite these relevant findings, this study has some limitations that should be acknowledged. Sampling was restricted to two wards of a single hospital, which may limit the generalizability of the findings to other healthcare environments. Moreover, no longitudinal clinical follow-up was conducted to explore associations between fungal exposure and patient outcomes. Future studies that expand the sampling scope and integrate clinical data will be essential to clarify the impact of hospital air mycobiota on vulnerable populations.

The detection of airborne *Aspergillus* species, some of which are emerging and cryptic and exhibit reduced antifungal susceptibility, highlights a critical risk in hospital environments. Elevated MICs for first-line antifungals and the presence of potentially toxigenic species signal the need for strengthened infection control policies, continuous air surveillance, diagnostic tools, and personal training. This study not only reveals the underestimated fungal diversity in hospital air but also highlights the pressing need for integrative approaches that couple environmental monitoring with clinical vigilance to mitigate the growing threat of antifungal resistance.

## Data Availability

No datasets were generated or analysed during the current study.
